# IL‐19 induced by IL‐13/IL‐17A in the nasal epithelium of patients with chronic rhinosinusitis upregulates MMP‐9 expression via ERK/NF‐κB signaling pathway

**DOI:** 10.1002/clt2.12003

**Published:** 2021-03-31

**Authors:** Xia Li, Jiancong Huang, Xiaohong Chen, Xiaoping Lai, Zizhen Huang, Yue Li, Shuaixiang Li, Lihong Chang, Gehua Zhang

**Affiliations:** ^1^ Department of Otorhinolaryngology The Third Affiliated Hospital of Sun Yat‐Sen University Guangzhou China

**Keywords:** chronic rhinosinusitis, IL‐19, MMP‐9, nasal epithelium

## Abstract

**Background:**

Tissue remodeling is a crucial characteristic of chronic rhinosinusitis (CRS). Imbalance between matrix metalloproteinases (MMPs) and tissue inhibitors of metalloproteinases (TIMPs) is crucial for the pathologic tissue remodeling in CRS. Elevation of interleukin (IL)‐19 or MMP‐9 levels in patients with CRS had been proven in previous studies. Here, we aimed to investigate the role of IL‐19 in mediating MMP‐9 expression in CRS.

**Methods:**

Nasal tissue samples were collected from 45 individuals having chronic rhinosinusitis with nasal polyps (CRSwNP), 24 CRS without nasal polyps (CRSsNP), and 17 controls. Expression of IL‐19, its receptors (IL‐20R1/IL‐20R2), and MMP‐9 were investigated using RT‐qPCR and Immunofluorescence (IF). Human nasal epithelial cells (HNECs) were stimulated by IL‐19; ERK phosphorylation, nuclear factor‐κB (NF‐κB) pathway activation, and MMP‐9 level were detected by RT‐qPCR, enzyme‐linked immunosorbent assay, western blot, and IF. We also explored the effect of type1/2/3 cytokines on IL‐19 production by RT‐qPCR, and western blot.

**Results:**

Expression levels of IL‐19, its receptors (IL‐20R1/IL‐20R2), and MMP‐9 were increased in nasal tissues from individuals with CRSwNP compared to those with CRSsNP as well as the controls. IL‐19 significantly elevated the production of MMP‐9 in HNECs. Furthermore, IL‐19 could activate the ERK and NF‐κB pathways, accompanied by increased MMP‐9 production in HNECs. Conversely, both ERK and NF‐κB inhibitors significantly attenuated the role of IL‐19 in MMP‐9 production. siRNA knockdown of IL‐20R1 suppressed ERK and NF‐κB pathway activation, thereby decreasing MMP‐9 expression. IL‐13 and IL‐17A were found to stimulate IL‐19 production in HNECs.

**Conclusion:**

IL‐19, promoted by IL‐13 and IL‐17A, contributes to the upregulation of secretion of the tissue remodeling factor MMP‐9 in patients with CRS.

## 1 INTRODUCTION

Chronic sinusitis (CRS) is a common disease, with prevalence of 8% in China and 12% in the United States.^1^, ^2^, ^3^ It is a considerable public health concern as well as a socioeconomic burden.^4^ Clinically, CRS is categorized into two types: CRS with (CRSwNP) or without nasal polyps (CRSsNP). Histologically, CRSsNP is predominantly characterized by basement membrane thickening and interstitial fibrosis. On the contrary, CRSwNP is characterized by stromal tissue edema with albumin deposition and pseudocyst formation.^5^ Moreover, CRSwNP is considered a heterogeneous disease, and based on the expression of different inflammatory cytokines in CRSwNP, it can be divided into different clusters. Clinical characteristics, such as treatment outcomes, asthma prevalence, and postoperative recurrence, are significantly different across the clusters.^6^, ^7^ Despite the variation across subtypes of CRSwNP, manifestation of tissue remodeling is similar, hence indicating different inflammatory cytokines to eventually lead to similar tissue remodeling pattern through common downstream factors or pathways in nasal polyps.

Tissue remodeling in CRS involves transformation of the tissue structure and extracellular matrix (ECM). Several elements involved in tissue remodeling include matrix metalloproteinases (MMPs), tissue inhibitors of metalloproteinases (TIMPs), and transforming growth factor (TGF)‐*β*. Imbalance between MMPs and TIMPs is considered to be the key factor for pathological tissue remodeling in CRS.^8^, ^9^ As a family of zinc‐dependent proteolytic enzyme, MMPs can degrade a variety of constituents of ECM and regulate remodeling.^10^ However, MMP‐1, MMP‐2, and MMP‐3 expression in patients with CRS is controversial, whereas several studies have reported MMP‐7 and MMP‐9 levels to be higher in cases with CRSwNP.^11^, ^12^, ^13^, ^14^ Overall, the detailed upstream modulation of MMPs promoting tissue remodeling in chronic sinusitis remains unclear.

IL‐19 is one of the members of IL‐20 cytokine subfamily, which also includes IL‐20, IL‐22, IL‐24, and IL‐26.^15^ The structures of IL‐20 subfamily receptor heterodimers are somewhat similar. IL‐19 needs to bind to a functional heterodimeric receptor IL‐20R1/IL‐20R2 for mediating its signal transduction.^16^ Studies concerning IL‐20 subfamily have shown IL‐20 to induce MMP‐3 in primarily cultured human disc cells^17^; IL‐20 had also been reported to significantly promote MMP‐9 production in bladder cancer cells.^18^ E. Pace *et al*.^19^ had found IL‐19 to be overexpressed in the epithelium of patients with CRSwNP; however, whether IL‐19 mediates MMP‐9 expression in CRS, and if so, by what mechanism, still remains unclear.

Recent studies have suggested tissue remodeling of CRS to occur concurrently with inflammation.^20^ Studies, principally in Chinese patients, have shown CRSsNP to be characterized by Type 1 inflammatory patterns while CRSwNP was characterized by mixed type two‐thirds inflammatory profiles.^21^, ^22^ Whether IL‐19 could serve as a common downstream factor of type 1/2/3 cytokines in nasal epithelia, resulting in the regulation of MMP‐9, remains unknown. This study aimed to confirm the role of IL‐19 in regulating MMPs expression and explore the effect of type 1/2/3 cytokines on IL‐19 production in Human nasal epithelial cells (HNECs) of patients with CRS.

## 2 MATERIALS AND METHODS

### 2.1 Patients and tissue samples

We diagnosed and classified CRS according to the EPOS 2012 guidelines.^23^ CRSwNP was further diagnosed by bilateral, endoscopically visualized polyps in middle meatus, which had been confirmed by postoperative pathology. Patients with fungal rhinosinusitis, cystic fibrosis, primary ciliary dyskinesia, or severe pulmonary and cardiac diseases were excluded from this study. Besides, minors and pregnant women were also excluded. None of the enrolled patients had histories of surgery or the use of corticosteroids or antibiotics within the month prior to surgery. Subjects did not have acuter infection within a month prior to the sampling. Polyps from patients diagnosed with CRSwNP were obtained along with uncinate process and sinus mucosal tissues from patients with CRSsNP; control tissues of the inferior turbinate or uncinate process were obtained from patients without sinonasal disease during other rhinologic surgeries, such as pituitary adenoma, orbital decompression or nasoseptal deviation surgery. A part of the samples was stored at −80°C, and subsequently used for studying histologic changes, RNA and protein isolation, and immunofluorescence. The rest was freshly submerged in PBS supplemented with 1% antibiotic‐antimycotic (Invitrogen) for subsequent HNECs culture. More details can be found in the Supporting Information Material.

### 2.2 Human nasal epithelial cells culture

Human nasal tissues derived from patients with CRSwNP and CRSsNP were cut into 1 × 1‐mm fragments, digested in dispase II (50 mg/mL, Sigma‐Aldrich) overnight at 4°C, followed by further digestion in trypsin for 15 min at 37°C; the digestion was terminated by adding complete Dulbecco's modified eagle medium (DMEM; 90% DMEM containing 10% fetal bovine serum). The digested nasal tissue pieces were then filtered by a 100‐μm cell strainer to obtained cells. Cells were then suspended in complete DMEM and cultured in an incubator for 30 min to remove fibroblasts. Then the obtained HNECs were cultured in bronchial epithelial growth medium (BEGM; Lonza) added with 1% antibiotic‐antimycotic (Invitrogen) at a density of (3–5) × 10^5^ cells/cm^2^ at 37°C in an atmosphere of 5% CO_2_ and 95% relative humidity. BEGM was refreshed every 2 days, after 10–14 days incubation, HNECs were stimulated and collected for subsequent experiments.

### 2.3 Real‐time quantitative polymerase chain reaction

Real‐time quantitative polymerase chain reaction (RT‐qPCR) was performed, as described previously, to detect the mRNA level of target genes.^24^ More details can be found in Supporting Information Material.

### 2.4 Enzyme‐linked immunosorbent assay

Supernatant of HNECs was collected and examined by MMP‐9 ELISA kits (CUSABIO). All procedures followed the manufacturers' instructions.

### 2.5 Western blotting

Western blotting was performed as reported previously.^13^ The polyvinylidene fluoride membranes were first incubated with primary antibodies MMP‐9 (1:1000; Abcam), pERK, ERK, pIκB*α*, IκB*α* (1:1000; Cell Signaling Technology), and glyceraldehyde 3‐phosphate dehydrogenase (1:3000; PeproTech) overnight at 4°C separately, and then incubated with horseradish peroxidase‐conjugated secondary antibody (Bioworld). Color development was achieved by ECL reagents. More information can be found in Supporting Information Material.

### 2.6 Immunofluorescence staining and confocal microscopy

The frozen sections from human nasal tissue were blocked with 10% goat serum for 30 min and stained first with IL‐19 (1:50; Abcam) and MMP‐9 antibodies (1:50; Abcam) overnight at 4°C, separately. HNECs were fixed with 4% paraformaldehyde, blocked in 1% bovine serum albumin for 30 min, and then stained first with MMP‐9 or p65 antibodies (1:500; Cell Signaling Technology) overnight at 4°C. Subsequently, the frozen tissue sections and HNECs were incubated with Alexa Fluor 594‐/or 488‐conjugated secondary antibodies (1:1000; Invitrogen) for 1 h at room temperature. Finally, the slices were stained with 4′,6‐diamidino‐2‐phenylindole (1:30,000; Biolegend) to mark the nuclei, and sealed with antifade mountant (Invitrogen). IF was examined at ×400 magnification under a microscope (Leica), or at ×630 magnification in a confocal microscope (Carl Zeiss).

### 2.7 siRNA transfection and ERK and nuclear factor‐κB pathway inhibition

Human IL‐20R1 siRNA (5′‐CUUACACUGUGCAGUAUUUUU‐3′) was synthesized by IGE Biotechnology company. For siRNA transfection, HNECs were transfected for 48 h using Lipofectamine™ 3000 Reagent (Invitrogen), in line with the recommended protocol. Subsequently, HNECs were stimulated with 100 ng/mL IL‐19 (or without) for 24 h. For pathway inhibition, ERK and nuclear factor‐κB (NF‐κB) pathways were suppressed by inhibitors PD98059 or BAY 11‐7082 (MedChem Express) for 1 h, respectively. Similarly, HNECs were stimulated with IL‐19 for 24 h.

### 2.8 Statistical analysis

IBM SPSS 20 (SPSS) was used for statistical analyses. When data were normally distributed, they were presented as mean ± *SEM*; otherwise, they were presented as median (25–75 percentiles). One‐way analysis of variance or Kruskal–Wallis test was applied to analyze significance across groups, for comparative study, Student's *t* or Mann–Whitney *U* test (two‐tailed) was then applied for comparisons across groups. Paired Student's *t* or Wilcoxon matched‐pair signed rank test was used when suitable. *p* < 0.05 was regarded as significant.

## 3 RESULTS

### 3.1 Increased expression of IL‐19, its receptors (IL‐20R1/IL‐20R2), and MMP‐9 in nasal mucosa of patients with CRSwNP

**FIGURE 1:**
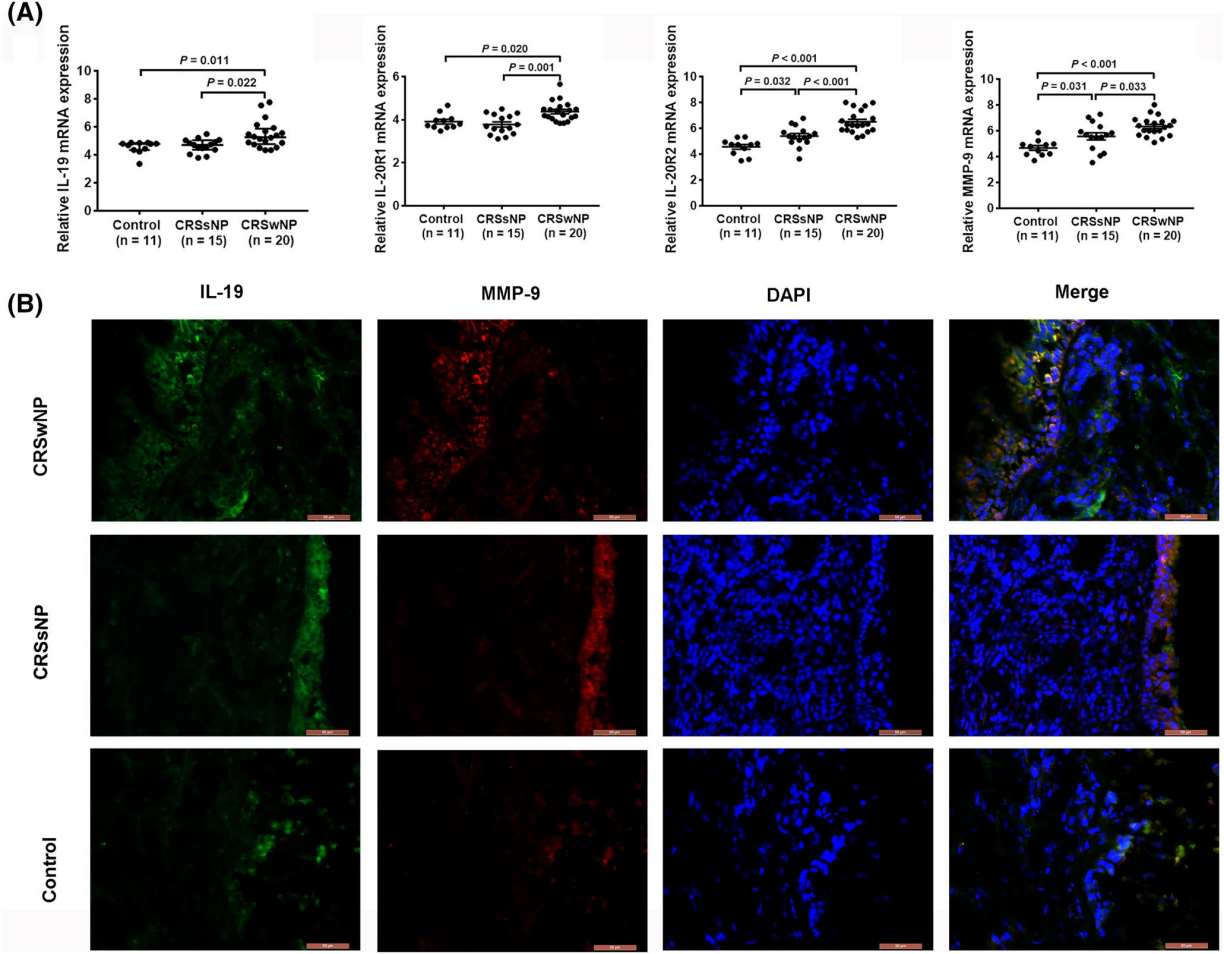
Increased expression of IL‐19, its receptors (IL‐20R1/IL‐20R2), and MMP‐9 in nasal mucosa of patients with CRSwNP. (A) RT‐qPCR results; relative mRNA levels of IL‐19, its receptors (IL‐20R1/IL‐20R2), and MMP‐9 (CRSwNP 20, CRSsNP 15, Control 11; IL‐19 are expressed as median [IQRs]; IL‐20R1, IL‐20R2, and MMP‐9 are expressed as mean ± *SEM*]. (B) Nuclei, IL‐19, and MMP‐9 are stained in blue, green, and red by immunofluorescence, respectively (magnification, ×400). *p < 0.05, **p < 0.01,***p < 0.001. CRSwNP, chronic rhinosinusitis with nasal polyps; IL, interleukin; MMP, matrix metalloproteinase; RT‐qPCR, real‐time quantitative polymerase chain reaction

Tissues collected from patients with CRSwNP and CRSsNP, and from controls, were examined for IL‐19, its receptors, and MMP‐9 expression by RT‐qPCR. As expected, IL‐19, its receptors (IL‐20R1/IL‐20R2), and MMP‐9 mRNA expression levels were significantly higher in patients with CRSwNP than in CRSsNP and control subjects**.** Besides, IL‐20R2 and MMP‐9 mRNA expression was increased in patients with CRSsNP than those in control subjects (Figure 1A). IF double staining was used to further evaluate the probable interaction between IL‐19 and MMP‐9. IL‐19 was mainly found co‐localized with MMP‐9 in the epithelial cells of CRS mucosa (Figure 1B). The above results suggested functional correlation of IL‐19 with MMP‐9 in the mucosal tissue of patients with CRS.

### 3.2 IL‐19 induced MMP‐9 expression in HNECs

**FIGURE 2:**
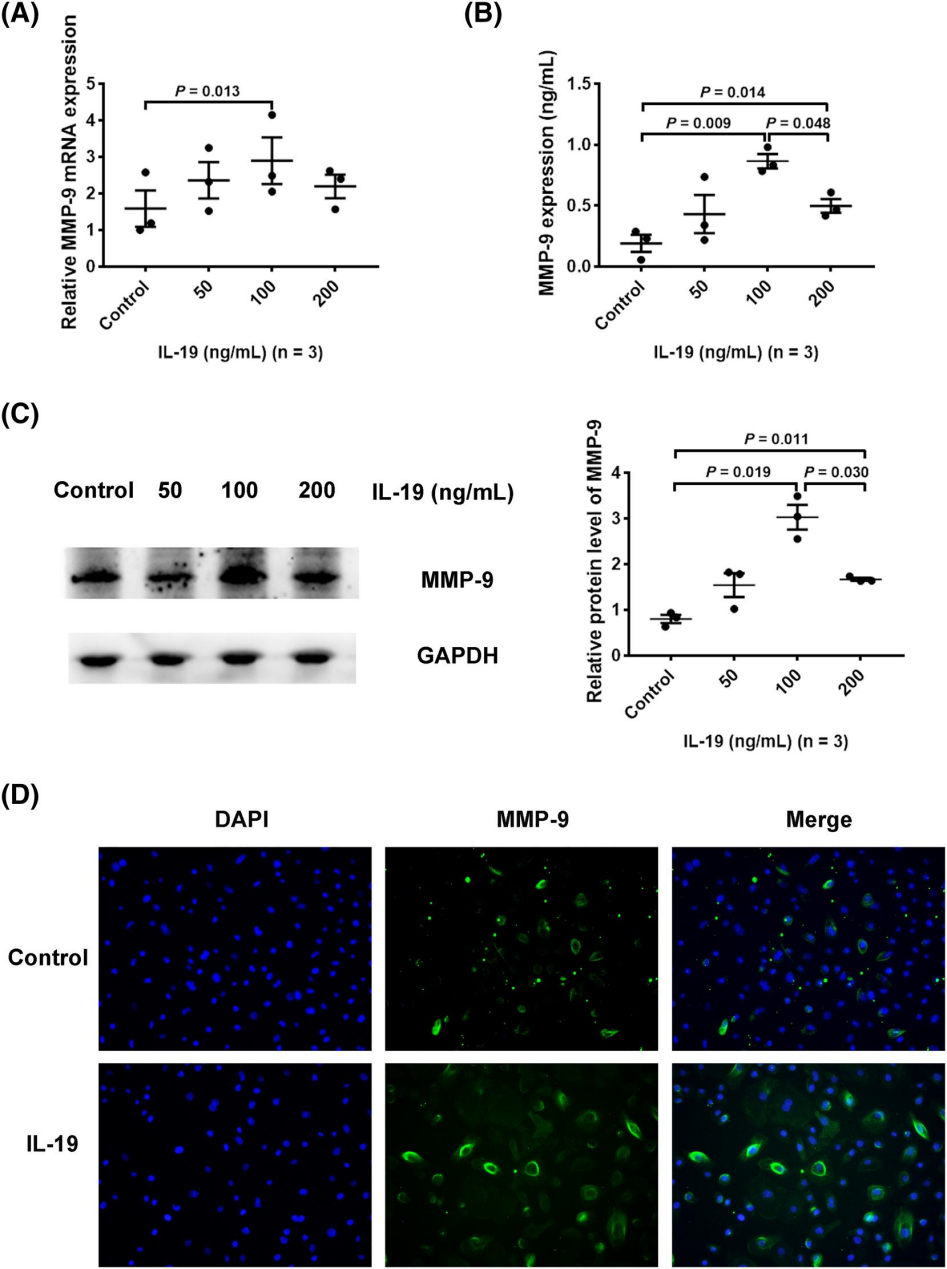
IL‐19‐induced MMP‐9 expression in HNECs. HNECs were incubated with gradient concentrations of IL‐19 for 24 h. (A, C) HNECs were collected to measure transcription and protein levels of MMP‐9 (*n* = 3). (B) HNEC supernatants were collected to measure MMP‐9 release by ELISA (*n* = 3). (D) HNECs were collected for immunofluorescence staining to detect MMP‐9 protein.*p < 0.05, **p < 0.01, mean ±*SEM*. ELISA, enzyme‐linked immunosorbent assay; HNEC, human nasal epithelial cell; IL, interleukin; MMP, matrix metalloproteinase

Considering the colocalization of IL‐19 and MMP‐9 in the mucosa of patients with CRS, we next explored whether IL‐19 could elevate MMP‐9 secretion in HNECs. HNECs were treated with IL‐19 (0–200 ng/mL) for 24 h. Thereafter, RT‐qPCR was used to assess mRNA levels while enzyme‐linked immunosorbent assay, western blotting, and immunofluorescence were applied to evaluate protein expression. As expected, both mRNA and protein expression of MMP‐9 were elevated in response to IL‐19 (Figure 2A–D); the optimal concentration of IL‐19 was 100 ng/mL.

### 3.3 IL‐19 promoted MMP‐9 expression *via* NF‐κB pathway in HNECs

**FIGURE 3:**
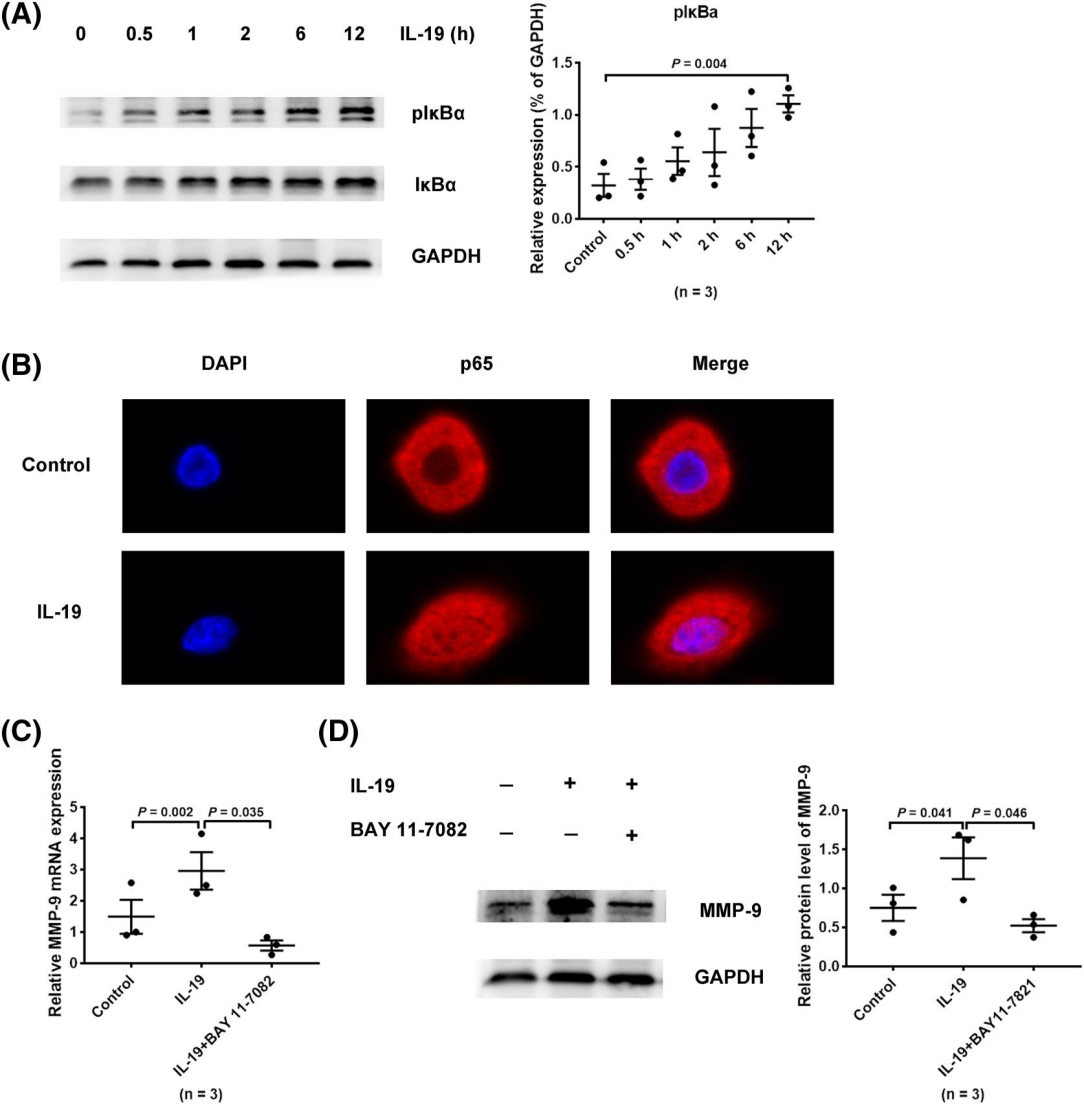
IL‐19 promoted MMP‐9 expression via NF‐κB pathway in HNECs. HNECs were treated with IL‐19 and collected at gradient time (0–12 h). (A) Western blotting detected the phosphorylation levels of IκBα (*n* = 3). (B) HNECs were incubated with IL‐19 for 24 h, p65 nuclear translocation was detected by confocal microscopic analysis. (C, D) HNECs were pretreated with BAY 11‐7082 (5 μM) for 1 h and subsequently treated with IL‐19. HNECs were collected to measure transcription and protein levels of MMP‐9 by RT‐qPCR and western blot, respectively (*n* = 3). *p < 0.05, **p < 0.01, mean ± *SEM*. HNEC, human nasal epithelial cell; IL, interleukin; MMP, matrix metalloproteinase; RT‐qPCR, real‐time quantitative polymerase chain reaction

The MMP‐9 gene promoter includes several transcription factors, and NF‐κB was verified as a binding site.^25^ To confirm the signaling pathways that induce up‐regulated MMP‐9 production in HNECs in response to IL‐19, first we evaluated whether IL‐19 could promote the activation of NF‐κB in HNECs. HNECs were treated with IL‐19 at optimal concentrations (100 ng/mL), and phosphorylation level of IκB*α* protein was measured by western blot while p65 nuclear translocation was detected by confocal microscopy. Results suggested treatment with IL‐19 to elevated the phosphorylation of IκB*α* effectively in HNECs (Figure 3A). Besides, compared to untreated HNECs, the DNA‐binding capacity of NF‐κB factor p65 was increased in response to IL‐19 treatment (Figure 3B). To further determine whether IL‐19 upregulated MMP‐9 by activating NF‐κB pathway, HNECs were preincubated with BAY 11‐7082 (IκB*α*‐specific inhibitor) for 1 h before IL‐19 stimulation. When BAY 11‐7082 was used, both MMP‐9 mRNA and protein, promoted by IL‐19, were attenuated significantly, as seen in RT‐qPCR and western blot, respectively (Figure 3C,D). Collectively, therefore, these findings confirmed NF‐κB pathway to participate in IL‐19‐promoted MMP‐9 production in HNECs.

### 3.4 IL‐19 upregulated MMP‐9 expression via ERK pathway in HNECs

**FIGURE 4:**
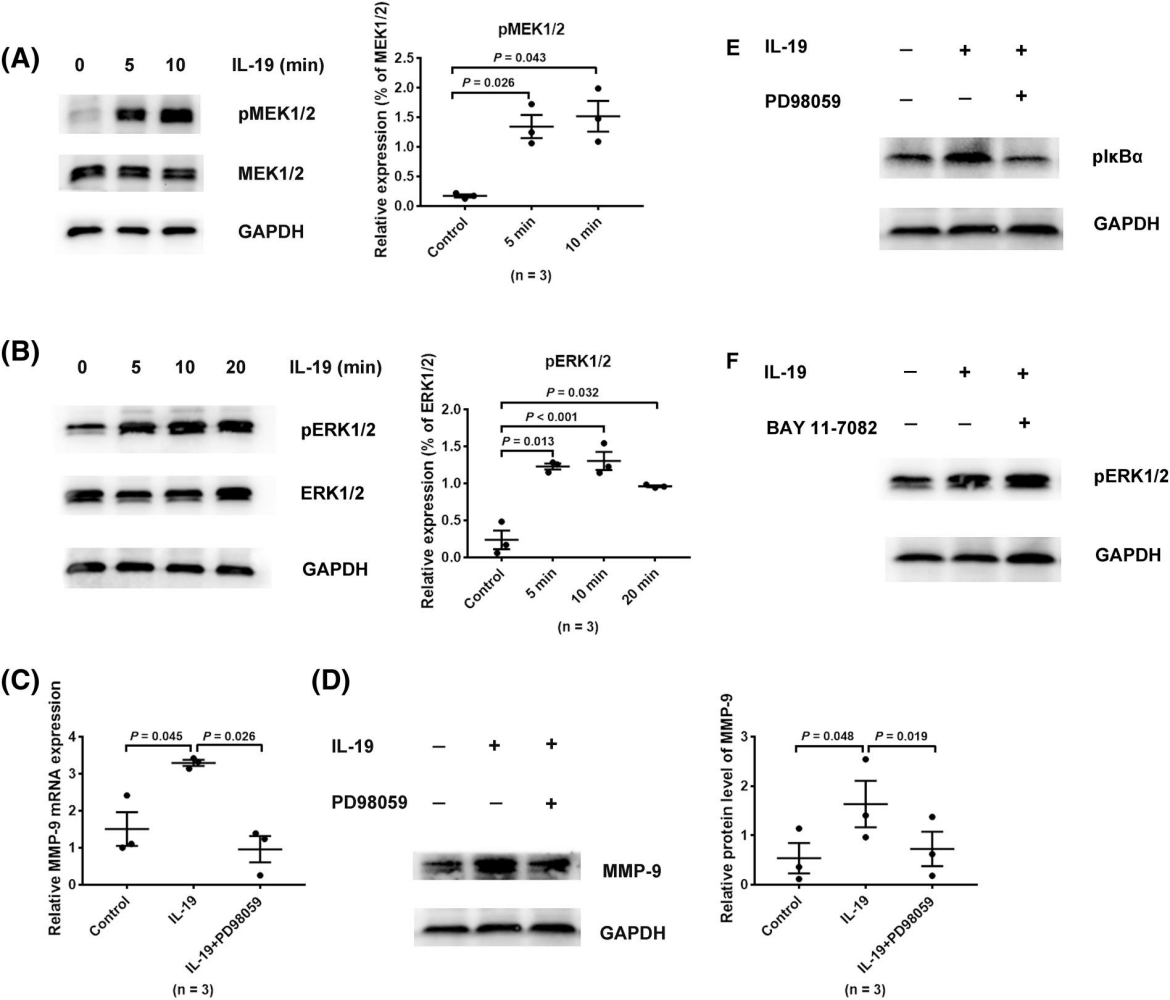
IL‐19 upregulated MMP‐9 expression via ERK pathway in HNECs. (A, B) HNECs were treated with IL‐19 and collected at gradient time (0–20 min). Thereafter, western blotting was performed to measure the phosphorylation of MEK1/2 and ERK1/2 (*n* = 3). (C, D) HNECs were preincubated with PD98059 (20 μM) for 1 h and subsequently treated with IL‐19. They were subsequently collected to measure transcription (*n* = 3) and protein (*n* = 3) levels of MMP‐9 by RT‐qPCR and western blot, respectively. (E, F) HNECs were pretreated with PD98059 (20 μM) or BAY 11‐7082 (5 μM) for 1 h before being stimulated with IL‐19, and western blotting was performed to measure the phosphorylation of IκBα or ERK1/2, respectively. *p < 0.05, **p < 0.01, ***p < 0.001, mean ± *SEM*. HNEC, human nasal epithelial cell; IL, interleukin; MMP, matrix metalloproteinase; RT‐qPCR, real‐time quantitative polymerase chain reaction

Since earlier studies had demonstrated the NF‐κB pathway to be regulated by ERK signaling,^10^ we investigated the effect of IL‐19 on upstream signaling molecules in MAPK signaling. HNECs were stimulated by IL‐19 and collected at a time gradient (0–20 min). Stimulation of HNECs with IL‐19 induced an effective activation of MEK1/2 (Figure 4A) and ERK1/2 (Figure 4B), as seen in western blot. To determine whether the activation of ERK signaling was liable for IL‐19‐promoted MMP‐9 production, HNECs were preincubated with PD98059 (MEK1/2 inhibitor) followed by IL‐19 stimulation. However, preincubation with PD98059 attenuated the IL‐19‐promoted MMP‐9 expression in HNECs (Figure 4C,D)**,** thus indicating the participation of ERK signaling in IL‐19‐induced MMP‐9 production in HNECs.

Since the results suggested inhibition of both ERK and NF‐κB to block the IL‐19‐induced MMP‐9 production in HNECs, we next assessed whether ERK signaling included the pathways that resulted in the combined activation of NF‐κB in IL‐19‐treated HNECs. HNECs were treated with PD98059 or BAY 11‐7082 in presence of IL‐19, and phosphorylation of IκB*α* and ERK1/2 was checked by western blotting. Results indicated pretreatment of HNECs with PD98059 to attenuate IL‐19‐promoted phosphorylation of IκB*α* while preincubation with BAY 11‐7082 had no effect on IL‐19‐promoted activation of ERK1/2 in HNECs (Figure 4E). These findings demonstrated the participation of ERK signaling in IL‐19‐promoted MMP‐9 production by activating NF‐κB in HNECs.

### 3.5 IL‐19 stimulated MMP‐9 production by binding to its receptor in HNECs

**FIGURE 5:**
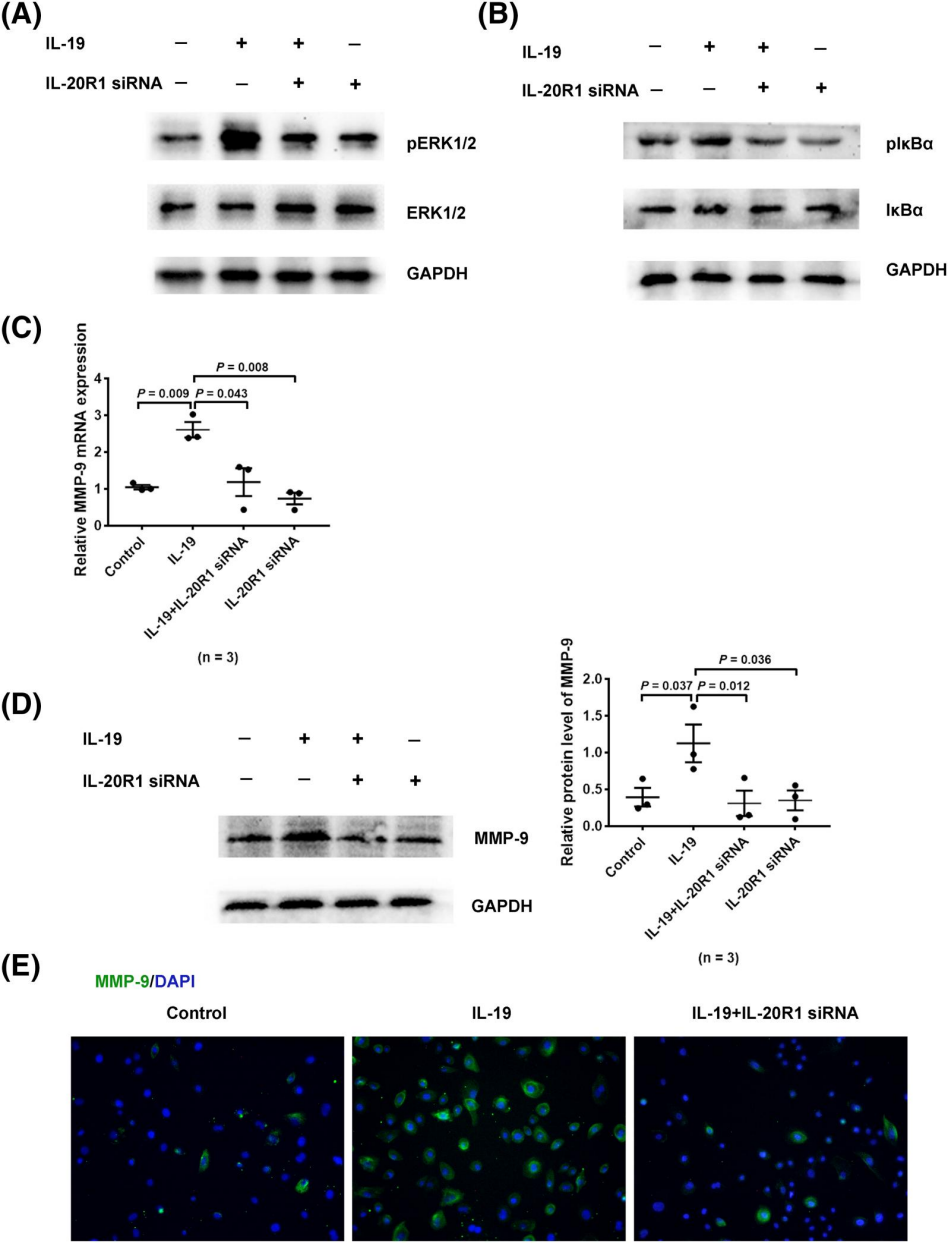
IL‐19 stimulated MMP‐9 production by binding to receptor in HNECs. (A, B) HNECs were transfected with IL‐20R1 siRNA for 48 h and subsequently incubated with IL‐19 for 24 h. They were then collected and western blotting performed to measure the phosphorylation of ERK1/2 (*n* = 3) and IκBα (*n* = 3). (C–E) MMP‐9 expression was measured by RT‐qPCR (*n* = 3), western blot (*n* = 3), and immunofluorescence. *p < 0.05, **p < 0.01, ***p < 0.001, mean ± *SEM*. HNEC, human nasal epithelial cell; IL, interleukin; MMP, matrix metalloproteinase; RT‐qPCR, real‐time quantitative polymerase chain reaction

IL‐19 transmits its signal by binding to its receptors (IL‐20R1/IL‐20R2 receptor).^26^ To determine whether IL‐19 stimulated MMP‐9 secretion through IL‐20R1 receptor, HNECs were pretreated with IL‐20R1 siRNA for 48 h before being stimulated by IL‐19. We found the interference of IL‐20R1 to significantly attenuate the activation of ERK1/2 and IKB*α* in HNECs in response to IL‐19 (Figure 5A,B). Besides, si‐IL‐20R1 significantly inhibited the IL‐19‐promoted MMP‐9 expression in HNECs, as seen by RT‐qPCR (Figure 5C), western blotting (Figure 5D), and immunofluorescence (Figure 5E). These findings together demonstrated IL‐19 to possibly mediate ERK and NF‐κB pathway activation, and MMP‐9 production in HNECs by binding to IL‐20R1.

### 3.6 IL‐13 and IL‐17A upregulated IL‐19 production in HNECs

**FIGURE 6:**
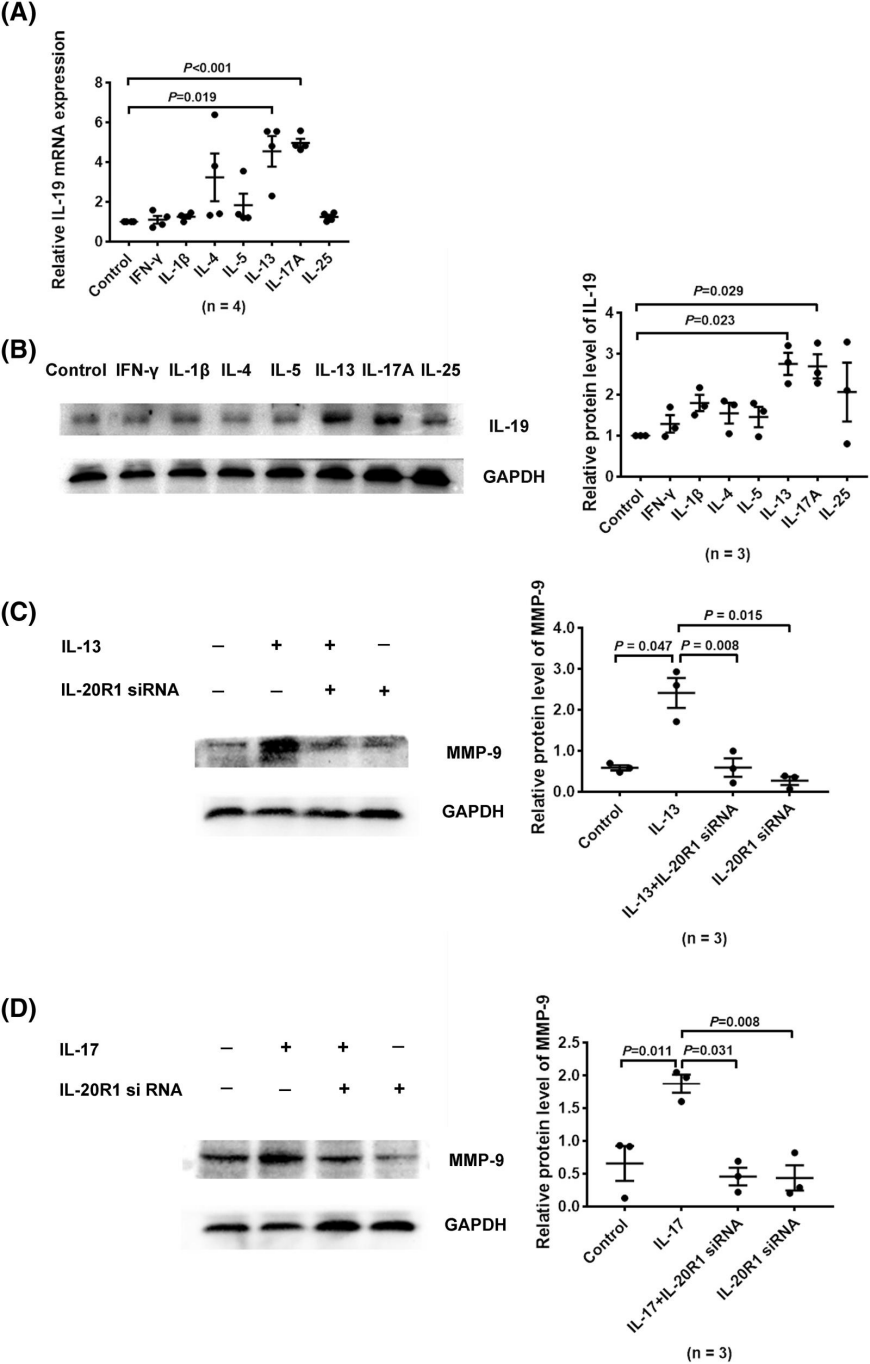
IL‐13 and IL‐17A stimulated IL‐19 production in HNECs. HNECs were incubated with Type 1 cytokines (IFN‐*γ*, IL‐*β*), Type 2 cytokines (IL‐4, IL‐5, and IL‐13), Type 3 cytokines (IL‐17A), and IL‐25 at a concentration of 20 ng/mL. (A) After 6‐h incubation, HNECs were collected to measure transcription levels of MMP‐9 (*n* = 4). (B) After 12‐h stimulation, proteins were extracted and measured by western blotting (*n* = 3). (C, D) HNECs were transfected with IL‐20R1 siRNA for 48 h and subsequently stimulated with IL‐13 or IL‐17A for 24 h. HNECs were collected to measure MMP‐9 expression, and analyzed using quantitative western blot (*n* = 3). *p < 0.05, **p < 0.01, ***p < 0.001, mean ± *SEM*. HNEC, human nasal epithelial cell; IL, interleukin; MMP, matrix metalloproteinase; RT‐qPCR, real‐time quantitative polymerase chain reaction.

To determinate whether the expression of IL‐19 was regulated by cytokines, HNECs were treated with Type 1 cytokines (IFN‐*γ*, IL‐1*β*), Type 2 cytokines (IL‐4, IL‐5, and IL‐13), Type 3 cytokines (IL‐17A), and IL‐25. RNA was collected, after 6‐h stimulation, for conducting RT‐qPCR, and proteins were extracted after 24‐h treatment for western blot. Only IL‐13 and IL‐17A were found to promote IL‐19 expression in RT‐qPCR (Figure 6A) and western blot (Figure 6B). In order to confirm whether IL‐13 and IL‐17A could elevate MMP‐9 secretion bypass IL‐19 production, HNECs were pretreated with IL‐20R1 siRNA for 48 h before stimulation with IL‐13 and IL‐17A. The protein levels of MMP‐9 were effectively increased in response to IL‐13 and IL‐17A. Moreover, the promoting effect of IL‐13 and IL‐17A on MMP‐9 production could be partly suppressed by IL‐20R1 siRNA (Figure 6C,D). Collectively, our data showed IL‐13 and IL‐17A cytokines to be capable of promoting the secretion of IL‐19 in HNECs.

## 4 DISCUSSION

One of the main events in tissue remodeling is the degradation of ECM components by proteolytic enzymes.^27^ Insufficient suppression of MMP‐9 by TIMP‐1 in polyp could lead to degradation of ECM, and hence, pseudocyst formation.^28^ However, TGF‐*β*1 can promote TIMP‐1 expression, and elevated levels of TGF‐*β*1 and TIMP‐1, relative to MMP‐9, may account for the principal fibrosis observed in CRSsNP.^29^ In our previous study^12^ and the current one, we demonstrated MMP‐7 and MMP‐9 expression in CRSwNP to be elevated compared to that in CRSsNP and the control group, which was consistent with other previous reports.^11^, ^14^, ^29^ MMPs could be an important treatment target for chronic rhinosinusitis. As one of the MMPs inhibitors, doxycycline has been demonstrated to effectually diminish MMP‐9 expression in nasal secretions, eventually shrinking the size of polyps.^30^ Studies have also shown high concentrations of MMP‐9 after functional endoscopic sinus surgery (FESS) to be related to bad treatment outcome.^31^ Doxycycline‐releasing sinus stents have been shown to effectively reduce nasal MMP‐9 concentrations and improve therapeutic effect in patients post FESS.^32^ Therefore, the upstream regulators of MMP‐9 in CRS would be worth studying.

Recent reports have indicated IL‐20 subfamily to participate in many inflammatory diseases, liver fibrosis, rheumatoid arthritis, psoriasis, and asthma.^33^, ^34^, ^35^, ^36^ Our previous studies had shown IL‐19 to be involved in mucin production, a participant in tissue remodeling in CRS.^37^ In the present study, mRNA levels of IL‐19, its receptors (IL‐20R1/IL‐20R2), and MMP‐9 were found elevated in CRSwNP. Although smoking and atopy may be considered to affect results, the multiple linear regression proved that only grouping variable was responsible for change in IL‐19 mRNA expression in human nasal tissues in our study (Table S4). Additionally, IL‐19 was mainly seen to be colocalized with MMP‐9 in the epithelium of CRS mucosa. According to the above results, we speculated IL‐19 to possibly participate in the development and progression of tissue remodeling in CRS. Thereafter, we aimed to investigate how IL‐19 could conduce to the modulation of MMP‐9 production in HNECs. As expected, IL‐19 stimulation was observed to promote MMP‐9 production in HNECs at an optimal concentration of 100 ng/mL. Additionally, transfection of IL‐20R1 siRNA into HNECs effectively attenuated IL‐19‐promoted MMP‐9 production. The function of IL‐19 might manifest after combining with IL‐20R1 receptor, thus resulting in MMP‐9 overexpression and ECM destruction. This finding was in line with studies concerning the function of IL‐19 depending on its binding to a functional heterodimeric receptor IL‐20R1/IL‐20R2.^26^ In contrast, IL‐19 had no effect on the expression of MMP‐7 in HNECs (Figure S1).

Till date, only a few studies have been reported about the specific mechanism of MMP‐9 modulation in CRS. Previous studies had demonstrated IL‐20 subfamily members to activate the signal transducer and activator of transcription (STAT), Janus kinase, and mitogen‐activated protein kinase (MAPK) signaling pathways.^15^ As a subclass of MAPK pathways, the ERK signaling pathway is also activated by a sequence of phosphorylation events in response to extracellular stimuli, which then transmits extracellular signals that induce cellular proliferation, differentiation, and survival. After being activated, RAF kinases (MAPKKK) phosphorylate and activate the elements of MAPKK module MEK1/2, which then activate the MAPK protein kinase, ERK1/2. Once activated, ERK1/2 phosphorylates and connects various extracellular signals to cytoplasm or nucleus.^38^ In our current study, we demonstrated effective induction of phosphorylation of MEK1/2 and ERK1/2, modules of ERK pathway, and expression of MMP‐9 in HNECs by IL‐19. We also identified the participation of transcription factor NF‐κB in ERK‐regulated MMP‐9 production in IL‐19‐stimulated HNECs. These findings were similar to that reported in studies concerning atherosclerosis and bladder cancer. In reports regarding the pathogenesis of atherosclerosis, CD147 had been demonstrated to mediate MMP‐9 induction through ERK and the nuclear translocation of NF‐κB pathway in macrophage.^39^ IL‐20 could also induce bladder cancer cell migration by activating the ERK‐mediated NF‐κB pathway, and promoting MMP‐9 production subsequently.^18^ Regulatory components that exist in the MMP‐9 gene contain the binding site for NF‐κB, which has been proved to drive MMP‐9 production in macrophages, neutrophils, cardiomyocytes, fibroblasts, and bladder cancer cells.^18^, ^25^, ^40^, ^41^, ^42^ As a dimeric transcription factor (p50/p65), the classical NF‐κB acts as a pivotal inflammatory regulator and modulates various genes. Upon activation, NF‐κB translocates to the nucleus, after which the active subunit p65 promotes transcription of chemokines, cytokines, and adhesion molecules.^43^ We demonstrated the promotion of NF‐κB activation by IL‐19, which generated phosphorylation of IκB*α* and translocation of p65 subunit, accompanied by increased MMP‐9 expression in HNECs. Besides, this excessive expression of MMP‐9 could be eliminated by NF‐κB pathway‐specific inhibitor BAY 11‐7082. Furthermore, our study on the inhibitor PD98059 suggested the classical NF‐κB pathway, activated by IL‐19, to be mediated by ERK signaling.

Accumulating studies have shown inflammatory cytokines to be important components of the nasal environment of CRS, and to participate in tissue remodeling.^44^ Although macrophages, keratinocytes, fibroblasts, airway epithelial cells, and nasal epithelia have been demonstrated to be the cellular sources of IL‐19,^15^, ^19^ very few studies have focused on the stimulatory effects of cytokines on IL‐19 production, especially in CRS. However, IL‐4, IL‐13, and IL‐17A have been proven to perform stimulatory function in IL‐19 production in airway epithelia. Besides, IL‐13 and IL‐17A collectively act to further stimulate IL‐19 production through a STAT6‐dependent transcriptional pathway in airway epithelial cells.^45^ Similar to this study, we observed IL‐13 and IL‐17A to be capable of upregulating IL‐19 production in HNECs. Furthermore, the acceleration of MMP‐9 production by IL‐13 and IL‐17A could be partly suppressed by IL‐20R1 siRNA. Our study suggested IL‐13 and IL‐17A to possibly participate in the tissue remodeling of CRS by positively regulating the MMP‐9 expression through IL‐19 secretion in HNECs. It indicated that CRSwNP subgroups with different clinical characteristics might eventually cause similar tissue remodeling patterns by affecting IL‐19 expression. Our findings provide important insights into the pathogenesis of CRS and propose a potential therapeutic target to suppress pathological nasal tissue remodeling in future.

However, there are still some limitations in our study. Since our results were based on in *vitro* experiments so that the results cannot be directly transferred to the patients with CRS. In order to further validate the effect of IL‐19 on the tissue remodeling of CRS, animal model mimicking disease status of human CRS should be constructed first. Afterward, IL‐19 and anti‐IL‐19 monoclonal antibody should be given to animal model and tissue remodeling factors should be detected. Meanwhile, the method, frequency and duration of administration of IL‐19 and anti‐IL‐19 monoclonal antibody in animal model also need to be explored. If the function of IL‐19 in tissue remodeling is confirmed in CRS animal model, relevant clinical trials in CRS patients are still needed. In a word, to achieve better clinical perspectives as a therapeutic strategy, more researches about the effect of IL‐19 on CRS tissue remodeling are still needed in the future.

In conclusion, we demonstrated prominent expression of IL‐19 in nasal polyps of patients with CRSwNP, along with increased expression of tissue remodeling factor MMP‐9. We then focused on the role of IL‐19 in modulating MMP‐9 expression in HNECs. Based on our findings, we proposed the novel concept of IL‐19 up‐regulating MMP‐9 production by binding to IL‐20R1 in HNECs. Moreover, IL‐19 promoted MMP‐9 expression by stimulating the classical NF‐κB pathway, mediated by ERK signaling. Our results also suggested IL‐13 and IL‐17A to elevate IL‐19 expression in HNECs. Collectively, our findings illustrated the participation of IL‐19 (promoted by IL‐13 and IL‐17A) in tissue remodeling in patients with CRS.

## CONFLICT OF INTERESTS

The authors declare that there are no conflict of interests.

## AUTHOR CONTRIBUTIONS

Xia Li, Jiancong Huang, and Xiaohong Chen performed RT‐qPCR, western blot, and ELISA, and prepared the manuscript. Xiaoping Lai and Zizhen Huang performed immunofluorescence. Yue Li and Shuaixiang Li participated in sample collection. Gehua Zhang and Lihong Chang designed the study and prepared the manuscript.

## Supporting information

Supplementary Material
