# Supplementary material for: IL‐19 induced by IL‐13/IL‐17A in the nasal epithelium of patients with chronic rhinosinusitis upregulates MMP‐9 expression via ERK/NF‐κB signaling pathway
Source: Clin Transl Allergy. 2021 Mar 31;11(1):e12003. doi: 10.1002/clt2.12003 (PMC8099262; doi:10.1002/clt2.12003)
Supplement: Supplementary file 1 — Supplementary Material [file CLT2-11-e12003-s001.docx]

**Online supplementary**

**Materials and methods**

**2.3** | **Real-time quantitative PCR (RT-qPCR)**

Total RNA of HNECs was isolated using RNAiso Plus (TaKaRa Biotechnology, Dalian, Japan) and reverse-transcribed to cDNA with PrimeScript™ RT Master Mix kit (TaKaRa Biotechnology, Dalian, Japan). PCR was performed with an ABI 7500 FAST instrument (Foster City, CA, USA) using a SYBR Premix Ex Taq kit (TaKaRa Biotechnology, Dalian, Japan) with the primers (Invitrogen, Carlsbad, CA, USA) listed in supplement table S2. Relative quantification was performed by the 2-^∆∆CT^ method against control β2 microglobulin (β2M).

**2.5** | **Western blot**

Proteins were extracted from HNECs and subjected to 8%–10% SDS-PAGE. The separated proteins were transferred to a PVDF membrane (Millipore, Billerica, MA, USA), which was then blocked with 5% skim milk in TBS for 1 h. The membranes were first incubated with primary antibodies MMP-9 (1:1000; Abcam, Cambridge, UK), PERK, ERK, PIκBα, IκBα (1:1000; Cell Signaling Technology, Boston, MA, USA), and GAPDH (1:3000; Protech), overnight at 4 °C, and then with HRP-conjugated secondary antibody (Bioworld, Nanjing, China) for 1.5 h. Blots were developed using ECL reagents. Relative intensity of each band was normalized to that of GAPDH.

**2.9** | **TEER (transepithelial resistance)**

The TEER of HNECs was measured at day0, day5 and day14 by using an Epithelial Volt Ohm Meter (Millipore, Billerica, MA, USA). TEER was calculated by subtracting blank value. The total TEER (ohms·cm2) was presented by TEER measurement (ohms) × Area of a membrane (cm^2^).

**3.0** | **CCK8 assay**

Cell viability was measured by CCK-8 kit (Dojindo, Kumamoto, Japan). HNECs were seeded into 96-well plates at amount of 100 µl/well and incubated at 37°C in 5% CO_2_ incubator, after cytokines stimulation /or not, 10 µl CCK reagent was added to each well, HNECs were then cultured another 2 h. Optical density (OD) value of each well was detected at 450 nm by a microplate reader (Biotek, Winooski, Vermont, USA).

**Table S1. Characteristics and methods**

|  | Control | CRSsNP | CRSwNP | *P* value |
| --- | --- | --- | --- | --- |
| Total subject number | 17 | 24 | 45 | NA. |
| Gender, male/female | 12/5 | 15/9 | 31/14 | 0.824 |
| Age (y), as mean (SD) | 32.12 (12.03) | 42.17 (14.46) | 36.96 (16.13) | 0.069 |
| Atopy, N (%) | 4 (29.41) | 4 (16.67) | 12 (26.67) | 0.645 |
| Smoking, N (%) | 1 (5.88) | 3 (12.50) | 5 (11.11) | 0.776 |
| Aspirin sensitivity, N (%) | 0 | 0 | 0 | NA. |
| Asthma, N (%) | 0 | 0 | 0 | NA. |
| **METHODOLOGIES USED** | | | |  |
| Tissue mRNA | 11 | 15 | 20 |  |
| Tissue immunoﬂourescence | 6 | 4 | 5 |  |
| HNECs culture | 0 | 5 | 20 |  |

CRSwNP, chronic rhinosinusitis with nasal polyps; CRSsNP, chronic rhinosinusitis without nasal polyps; NA, not applicable; *P* <0.05 was considered statistically significant.

**Table S2. Primers for RT-qPCR analysis**

| **Gene** | **Sequence** |
| --- | --- |
| IL-19 | (F) 5'-GGCAATGTCAGGAACAGAGG-3' |
|  | (R) 5'-AGCGGAATAAGACAGCCTGA-3' |
| IL-20R1 | (F) 5'-GCTCAGCCTTCTGAGAAGCAGTG-3' |
|  | (R) 5'-CGCACAAATGTCAGTGGTTCTGAC-3' |
| IL-20R2 | (F) 5'-GCTGGTGCTCACTCACTGAAGGT-3' |
|  | (R) 5'-TCTGTCTGGCTGAAGGCGCTGTA-3' |
| MMP-9 | (F) 5'-AGACCTGGGCAGATTCCAAAC-3' |
|  | (R) 5'-CGGCAAGTCTTCCGAGTAGT-3' |
| β2M | (F) 5'-TACACTGAATTCACCCCCAC-3' |
|  | (R) 5'-CATCCAATCCAAATGCGGCA-3' |

MMP-9, matrix metalloproteinase-9; β2M, β2 microglobulin

**Table S3. Cytokines and inhibitors for HNECs stimulation**

| Stimuluses | Duration | Concentration | Company |
| --- | --- | --- | --- |
| IFN-γ | 6 h for RT-qPCR, 12 h for WB | 20 ng/mL | R&D |
| IL-1β | 6 h for RT-qPCR, 12 h for WB | 20 ng/mL | R&D |
| IL-4 | 6 h for RT-qPCR, 12 h for WB | 20 ng/mL | R&D |
| IL-5 | 6 h for RT-qPCR, 12 h for WB | 20 ng/mL | R&D |
| IL-13 | 6 h for RT-qPCR, 12 h for WB | 20 ng/mL | R&D |
| IL-17A | 6 h for RT-qPCR, 12 h for WB | 20 ng/mL | R&D |
| IL-25 | 6 h for RT-qPCR, 12 h for WB | 20 ng/mL | R&D |
| IL-19 | 24 h | 100 ng/mL | R&D |
| PD98059 | 25 h (1 h before IL-19 stimulation) | 20 μM | R&D |
| BAY 11-7082 | 25 h (1 h before IL-19 stimulation) | 5 μM | R&D |

WB: western blot

**Table S4. The multiple linear regression analysis of IL-19 mRNA expression**

| Variables | Unstandardized coefficients B | Standard  error | Standardized coefficients β | *P* value |
| --- | --- | --- | --- | --- |
| Constant | 4.582 | 0.223 |  |  |
| CRSwNP group | 1.022 | 0.288 | 0.614 | 0.001 |
| CRSsNP group | 0.112 | 0.293 | 0.064 | 0.703 |
| Smoking | -0.022 | 0.337 | -0.009 | 0.949 |
| Atopy | -0.392 | 0.279 | -0.202 | 0.169 |

CRSwNP, chronic rhinosinusitis with nasal polyps; CRSsNP, chronic rhinosinusitis without nasal polyps. The multiple linear regression was used to specify which variables (grouping, smoking, atopy) was responsible for change in IL-19 mRNA expression in human nasal tissues. Only CRSwNP group variable was proved to be an effect factor. *P* < 0.05 was considered significant.


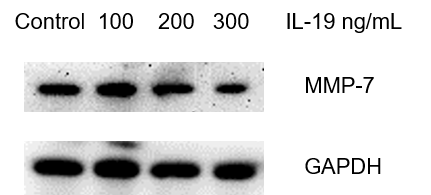


**Supplemental Fig. 1 IL-19 had no effect on the expression of MMP-7 in HNECs.**

HNECs were incubated with gradient concentrations of IL-19 (0, 100, 200, and 300 ng/mL) for 24 h. They were then collected to check the protein levels of MMP-7 by western blotting.

**Supplemental Fig. 2 TEER of HNECs during the culture period.**

HNECs were cultured and then the TEER of HNECs was measured at day0, day5 and day14 by Epithelial Volt Ohm Meter.

**Supplemental Fig. 3 type 1, 2, 3 cytokines, IL-19 and inhibitors stimulation had no effect on the** **cell viability of HNECs.**

HNECs were stimulated with IFN-γ, IL-1β, IL-4, IL-5, IL-13, IL-17A, IL-25 for 12 h, IL-19 for 24 h, BAY 11-7082 and PD98059 for 25 h. Then cell viability was measured by cck8 assay.
